# Resistance to Cell Death in Mucinous Colorectal Cancer—A Review

**DOI:** 10.3390/cancers13061389

**Published:** 2021-03-19

**Authors:** Emer O’Connell, Ian S. Reynolds, Deborah A. McNamara, John P. Burke, Jochen H. M. Prehn

**Affiliations:** 1 Department of Colorectal Surgery, Beaumont Hospital, Dublin 9, Ireland; emerpoconnell@rcsi.com (E.O.); ianreynolds@rcsi.ie (I.S.R.); deborahmcnamara@rcsi.ie (D.A.M.); johnburke@rcsi.ie (J.P.B.); 2 Department of Physiology and Medical Physics, Royal College of Surgeons in Ireland, Dublin 2, Ireland; 3 Department of Surgery, Royal College of Surgeons in Ireland, Dublin 2, Ireland; 4 Centre for Systems Medicine, Royal College of Surgeons in Ireland, Dublin 2, Ireland

**Keywords:** mucin, colorectal, apoptosis, chemo-resistance

## Abstract

**Simple Summary:**

Mucinous colorectal cancer is characterised by abundant mucin glycoprotein production and is associated with resistance to chemotherapy-based treatments. This review outlines mechanisms for cell death resistance present in mucinous colorectal cancer including glycoprotein interactions with cell survival and cell apoptotic pathways. Further mechanisms are explored, including alterations in the expression of chemotherapy metabolism and resistance genes. This review identifies directions for future investigation of mucinous colorectal cancer and novel treatment strategies.

**Abstract:**

Mucinous colorectal cancer (CRC) is estimated to occur in approximately 10–15% of CRC cases and is characterized by abundant extracellular mucin. Mucinous CRC is frequently associated with resistance to apoptosis. Inferior prognosis is observed in mucinous CRC, particularly in rectal cancer and metastatic cases. Mucins are heavily glycosylated secretory or transmembrane proteins that participate in protection of the colonic epithelium. MUC2 overexpression is a hallmark of mucinous CRCs. Mucinous CRC is associated with KRAS and BRAF mutation, microsatellite instability and the CpG island methylator phenotype. Mutations of the *APC* gene and *p53* mutations which are characteristic non-mucinous colorectal adenocarcinoma are less common in mucinous CRC. Both physical and anti-apoptotic properties of mucin provide mechanisms for resistance to cell death. Mucin glycoproteins are associated with decreased expression of pro-apoptotic proteins, increased expression of anti-apoptotic proteins and increased cell survival signaling. The role for BCL-2 proteins, including BCL-X_L_, in preventing apoptosis in mucinous CRC has been explored to a limited extent. Additional mechanisms opposing cell death include altered death receptor expression and altered mutation rates in genes responsible for chemotherapy resistance. The roles of alternate cell death programs including necroptosis and pyroptosis are not well understood in mucinous CRC. While the presence of MUC2 is associated with an immunosuppressive environment, the tumor immune environment of mucinous CRC and the role of immune-mediated tumor cell death likewise require further investigation. Improved understanding of cell death mechanisms in mucinous CRC may allow modification of currently used regimens and facilitate targeted treatment.

## 1. Introduction

**Figure 1:**
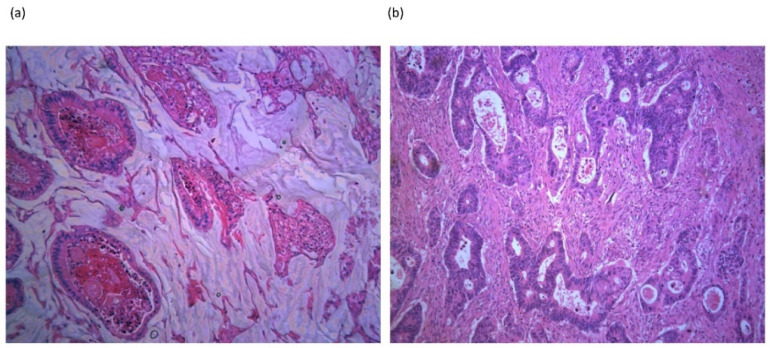
Representative histological slides of: (**a**) mucinous colorectal adenocarcinoma displaying abundant extracellular mucin in a 61 year old male patient with a pT4N2 mucinous rectal tumor. (**b**) Non-mucinous colorectal adenocarcinoma in a 67 year old female patient with a pT3N1 rectal tumor. H&E-stained section, 10× magnification, imaged with Leica DM 4000B microscope (Leica Microsystems, Mannheim, Germany).

Colorectal cancer (CRC) is a major health challenge: the global burden of CRC is expected to increase by 60% to more than 2.2 million new cases and 1.1 million deaths by 2030 [1]. Mucinous CRC is estimated to occur in approximately 10–15% of CRC cases and is characterized by abundant extracellular mucin that comprise of at least 50% of the tumor volume [2] (Figure 1). Mucinous CRCs occur more frequently in the proximal colon, occur at a younger age, and have a slight female predominance compared to non-mucinous CRC [3,4,5]. Mucinous CRCs tend to be larger than non-mucinous CRCs and are more commonly diagnosed at an advanced stage [4,5]. The molecular characteristics of mucinous CRC suggest that mucinous tumors develop and progress through different molecular pathways compared with non-mucinous CRCs.

## 2. Molecular Characteristics of Mucinous Colorectal Cancer

Sporadic CRC develops through a well-recognized pathway in approximately 85% of cases with accumulation of mutations in oncogenes and tumor suppressor genes leading to chromosomal instability (CIN). Mutations of the *APC* gene which are characteristic of chromosomal instability are less common in mucinous CRC [6]. *p53* mutations are common in tumors with chromosomal instability but less common in mucinous CRC [7]. Alternate pathways for CRC tumorigenesis are described including promotor methylation of the *MLH1* gene resulting in sporadic microsatellite instability [8], and also aberrant methylation and silencing of tumor suppressor genes termed epigenetic instability [9]. A meta-analysis of 46 studies describing 17,746 patients by Reynolds et al. in 2019 clarified molecular associations of mucinous CRC [10]. Mucinous CRC is associated positively with *KRAS* and *BRAF* mutation, microsatellite instability (MSI) and the CpG island methylator phenotype (CIMP) and negatively associated with altered *p53* expression [10]. *BRAF* and *KRAS* are both components of the RAS/MAPK pathway, and activation of this pathway promotes cell division and reduces cell apoptosis [11]. Mutations in *KRAS* lead to an epidermal growth factor receptor-independent disturbance of the RAS/RAF/MAPK pathway, which regulates cell proliferation and survival and is a prognostic factor in CRC [12]. *BRAF* mutations are commonly reported in patients with mucinous CRC and are associated with an infiltrative pattern of tumor growth [12,13]. In addition to mutations in *KRAS* and *BRAF* affecting the RAS/RAF/MAPK pathway, increased mutations are also reported in the PI3K/AKT pathways in patients with mucinous CRC [12]. Aberrations in the phosphoinositide 3-kinase (PI3K) signaling pathway play a key role in the pathogenesis of numerous cancers by altering cellular growth, metabolism, proliferation, and apoptosis. Leystra et al. demonstrated in a mouse model that expression of a dominantly active form of PI3K protein results in the development of mucinous colon cancers [14]. MSI is observed at a higher frequency in mucinous CRC compared to non-mucinous CRC. Likewise, Lynch syndrome (LS)-associated CRCs have a high rate of mucinous histology; approximately 22–40% of LS-associated CRCs are mucinous [15]. These finding substantiate the concept of an alternative oncogenic mechanism leading to mucinous CRC tumorigenesis, different from the classic adenoma-carcinoma sequence [12].

## 3. Expression of Mucins in Mucinous Colorectal Cancer

Mucins are heavily glycosylated secretory or transmembrane proteins that participate in protection of the colonic epithelium and limit inflammatory responses at the interface with the colonic lumen [16]. The secreted mucins include MUC2, MUC5AC, MUC5B and MUC6 and form a mucous gel which lines epithelial surfaces [16]. The transmembrane mucins include MUC1, MUC4, MUC13 and MUC16 [16]. These mucins span the cell membrane allowing external glycosylated structures to contribute to the mucous layer and transmembrane domains to interact with intracellular processes. MUC2 is the major component of the two layers of intestinal mucus and is expressed in the healthy colonic epithelium [17]. MUC2 production is increased in the goblet cells of the proximal colon [18], a site where mucinous CRCs are observed with greater frequency. In addition, the expression of MUC5AC which is located on the same chromosome as MUC2 is increased in mucinous CRC compared to non-mucinous CRC [19,20]. The presence of MUC2 and/or MUC5AC in colorectal mucinous adenocarcinoma has been shown to be associated with proximal (right-sided) CRC location [21]. MUC2 overexpression is a hallmark of mucinous CRCs [11,12,22]. In contrast, expression of MUC2 is generally decreased in non-mucinous CRC [22]. Loss of MUC2 expression is associated with increased proliferation of intestinal epithelial cells in response to mucosal inflammation [23]. The inflammation and tumor suppression role of MUC2 may appear paradoxical when it is observed that MUC2 expression is increased in mucinous CRC. It is suggested that MUC2 overexpression in cancer may reflect the mucin forming nature of the cancer’s cells rather than the intrinsic action of mucin [16]. Additionally, mucin expression may be modified by epigenetic mechanisms in cancers or mucin production may be used as an immune evasion mechanism by cancer cells by masking the cell surface from immune surveillance [16] (see Table 1).

Expression of MUC2 is regulated by a number of factors in the colonic epithelium. Analysis of mucin-producing cell lines by Okudaira et al. demonstrated that methylation of the MUC2 gene promotor was significantly lower in mucinous CRC lines compared to non-mucinous and correlated with mucin protein expression [24]. Loss of functional *p53* is responsible for downregulation of MUC2 expression [25]. MUC2 expression is transcriptionally regulated by *p53* protein in several cell lines [26]. Regulation of MUC2 by *p53* is consistent with the observation of reduced *p53* mutations in mucinous carcinomas, where MUC2 expression is increased [25]. In contrast, high rates of *p53* mutation and low expression of MUC2 are observed in non-mucinous CRC [26]. Additionally, the mitogen-activated protein kinase (MAPK) signaling pathway is known to regulate MUC2 gene transcription [27]. MAPK signaling is upregulated in the setting of *KRAS* mutation and in the setting of chronic inflammation, both of which are common features of mucinous cancer [28].

## 4. Acellular Mucin Pools Following Neoadjuvant Chemoradiotherapy

The presence of mucin pools in resection specimens with complete pathological response (pCR) has been observed following neoadjuvant treatment of rectal cancers with non-mucinous features prior to treatment. The significance of acellular mucins pools in the setting of complete tumor regression and their implications for tumor staging and prognosis have been evaluated in the literature. Comparison of survival outcomes between cases of pCR with or without mucin pools demonstrated no survival difference in a number of retrospective studies [45,46,47,48]. Mucin pools were found in proctectomy specimens in 12% (12/297) of patients following long course neoadjuvant chemoradiotherapy (NACRT) in a retrospective analysis by Reynold’s et al. [49]. The presence of mucin pools was associated with pCR and improved overall survival (OS), suggesting they may represent a treatment response. Analysis of 118 cases of locally advanced rectal cancer (LARC) achieving pCR after NACRT were retrospectively reviewed by Sun et al. Acellular mucin pools were observed in 27% and no difference in 5-year OS was observed in those with or without mucin pools. However, in univariate analysis, patients with mucin pools had increased risk of distant metastases, although this observation was not maintained on multivariate analysis [50]. More recently, Zhang et al. evaluated 117 locally advanced rectal cancers achieving pCR after NACRT. Acellular mucin pools were identified in 23%. In multivariate analysis, the presence of acellular mucin pools showed no prognostic impact on OS or disease-free survival (DFS) [51]. Overall, the literature suggests that the presence of acellular mucin pools in post-treatment pCR does not appear to be a significant prognostic factor and may be indicative of tumor response to therapy. The presence of residual tumor cells in mucin pools requires further prognostic clarification and underlines the need for careful pathological evaluation. Under current AJCC 8th edition guidelines, acellular mucin following neoadjuvant treatment is advised to represent eradicated tumor and should not upstage tumors [52].

## 5. Apoptosis and Other Cell Death Signaling Pathways in Mucinous Colorectal Cancer

BCL-2 family proteins are key for the activation of the mitochondrial or intrinsic apoptosis pathway [30]. The role of the BCL-2 family of proteins has been explored to a limited extent in mucinous CRC. The importance of BCL-2 and its family members in cell survival, differentiation, and oncogenesis has been demonstrated extensively in multiple cancers [31]. Dysregulation of BCL-2 proteins occurs during tumor development and is responsible for cellular resistance to chemotherapy treatments [53]. BCL-2 family proteins are described based on their pro-apoptotic or anti-apoptotic function [40]. Early immunohistochemical analysis of mucinous and non-mucinous CRCs by Zhang et al. demonstrated no association between mucinous histology and BCL-2 staining [41]. A similar analysis by Contu et al. observed no association between BCL-2 expression and mucinous histology in rectal cancers [32]. However, recent IHC study by Bhardwaj et al. identified BCL-2 positivity more frequently in non-mucinous colorectal tumors compared to mucinous [54]. In addition, a large proportion of colorectal tumors with microsatellite instability contain frameshift mutations in the *Bax* gene, a major apoptosis effector. Analysis of *Bax* mutations and apoptotic indices in colorectal tumors showed that mutations in *Bax* were associated with proximal, poorly differentiated and mucinous CRC [55].

Caspases 3 and 7 are activated downstream of mitochondrial permeabilization during the intrinsic apoptosis pathway, but may also be activated through death receptors and other intracellular caspase activating platforms [30]. Inhibitor of apoptosis proteins (IAPs) are natural inhibitors of caspases [30]. BIRC7/Livin is an inhibitor of apoptosis protein (IAP) associated with suppression of apoptosis in CRC [56]. Faruk et al. recently explored the expression of proteins relevant for apoptosis including *p53*, BCL2, Livin and Annexin V in mucinous and non-mucinous CRC [57]. Tumor samples from 23 patients with mucinous CRC were compared with 69 non-mucinous CRCs with neoadjuvant FOLFOX chemotherapy administered in a subset of the cohort. Livin expression was significantly lower in mucinous CRC at baseline compared to non-mucinous CRC. A significant increase in the expression of Livin was observed in mucinous CRC following treatment with FOLFOX chemotherapy while FOLFOX suppressed Livin expression in non-mucinous CRC. A significant increase in Annexin V was observed in FOLFOX treated non-mucinous CRC compared to FOLFOX treated mucinous CRC indicating increased apoptotic activity. No significant difference was observed between the expression of *p53* or BCL-2 in mucinous versus non-mucinous CRC. Systematic studies that analyze the entire framework of apoptosis signaling pathways in mucinous CRC are now warranted.

In addition to apoptotic programmed cell death, alternate cell death mechanisms including necroptosis and pyroptosis are known to occur CRC and in patients with inflammatory bowel disease (IBD) [33]. However, the role for necroptosis and pyroptosis in cell death requires exploration in mucinous CRC.

## 6. Mechanisms of Apoptosis Resistance in Mucinous Colorectal Cancer: Studies Exploring the Biological Functions of Individual Mucin Glycoproteins

### 6.1. MUC1 Glycoprotein

**Figure 2:**
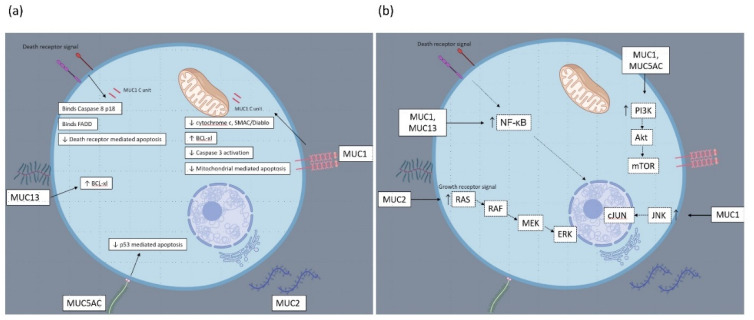
(**a**) Interaction of mucin glycoproteins with apoptosis proteins in colorectal cancer cells. (**b**) Cell survival mechanisms altered by expression of mucin glycoproteins in colorectal cancer cells.

Mucin glycoproteins are associated with the inhibition of apoptosis in both in vitro and in vivo models of CRC (see Figure 2). MUC1 is normally expressed on the apical borders of epithelial cells but is often overexpressed on the surface of cancer cells. Investigation in an in vitro colon cancer model used confocal microscopy to demonstrate that the MUC1-C-terminal intracellular domain (MUC1-C) localizes to the mitochondria [34]. Resistance to chemotherapy is frequently associated with defective intrinsic apoptosis and impaired release of mitochondrial cytochrome c. Release of cytochrome c in response to cisplatin was compared between colon cancer cells stably transfected with MUC1 versus empty vector cells. Expression of MUC1 significantly reduced cytochrome c release [34]. In addition, the release of SMAC/Diablo was attenuated in MUC1 expressing cells. Likewise, MUC1 attenuated activation of caspase 3 in response to cisplatin [34]. In vivo MUC1-expressing tumors had limited response to cisplatin treatment. Knockdown of MUC1 increased sensitivity to genotoxic drugs [34]. Further in vitro research from this group, although performed in a non-CRC model demonstrated further mechanisms for cell survival and apoptosis inhibition related to MUC1 [58]. MUC1 expression was found to influence PI3K-Akt signaling with increased in phosphor-Akt in MUC1 transfected cells. In addition, BCL-X_L_ expression was increased in MUC1 expressing cells. Treatment of MUC1 transfected and empty vector cells with an antimetabolite demonstrated decreased mitochondrial membrane potential in empty vector cells but not in MUC1 expressing cells. Activation of caspase 9 and caspase 3 cleavage activity was reduced in MUC1 expressing cells. The expression of BCL-X_L_ was not altered by use of a PI3K inhibitor, indicating BCL-X_L_ expression was independent of PI3K signalling [58].

Agata and colleagues demonstrated a critical role for MUC1 in response to death receptor signalling [35]. In vitro studies in mixed cell lines found that the MUC1 cytoplasmic domain binds directly to the caspase 8 p18 fragment and the FADD as a response to death receptor signalling. MUC1 was found to compete with caspase-8 for binding to FADD and blocked recruitment of caspase-8 to the death inducing signalling complex. This interaction could underlie impaired death receptor sginalling in malignant cells with overexpression of MUC1. This study supports the earlier work from this group who demonstrated that MUC1 blocked TRAIL-induced apoptosis of CRC cells [34]. Interestingly, resistance to death receptor signaling has been demonstrated in mucinous CRC tumor specimens. Analysis of CRC cases by Sheehan et al. indicated identified lower FasL expression in mucinous adenocarcinomas [36]. In addition, a small series from Koornstra et al. identified that DR4 expression was absent in sporadic mucinous MSI-high CRCs [59].

Within an in vitro CRC model, Chen et al. demonstrated that MUC1 reduces apoptosis to DNA damage through direct binding to JNK1 [37]. JNK (c-Jun N terminal kinase) is a member of the MAPK family. Treatment of MUC1-expressing colon cancer cells with cisplatin or doxorubicin induced phosphorylation of JNK1 and cJUN, with phosphorylation enhanced by ectopic expression of MUC1. MUC1 expression was found to significantly reduce PARP cleavage, an effect reversed in the presence of JNK inhibitor, indicating a critical role for JNK activation in MUC1 apoptosis resistance.

### 6.2. MUC2 Glycoprotein

Attenuation of MUC2 expression was found to significantly increase apoptosis in a mucinous colon cell line and a patient-derived xenograft model of mucinous peritoneal carcinomatosis in a study by Dilly et al. [60]. This group further explored treatments involving MUC2 attenuation to induce apoptotic cell death in mucinous CRC. The endoplasmic reticulum (ER) is a site for synthesis and protein folding including production of MUC2 [61]. Disruption of normal protein processing can cause overwhelming ER stress and trigger the unfolded protein response (UPR) while persistent ER stress will trigger cell death pathways [62]. Dilly and colleagues have hypothesized that mucinous CRC with high MUC2 production would be vulnerable to ER stress and ER-stress associated cell death [61]. Authors have demonstrated that basal markers of endoplasmic reticulum stress (ERS) are higher in mucinous tumors in in vitro and tumor explant models [61]. Treatment in vitro with ERS augmentation by orlistat and celecoxib induced apoptosis. Apoptosis was detected by TUNEL assay and detection of pro-apoptotic proteins BIM, NOXA, PUMA and activated cleaved caspase 3 and cleaved PARP by Western blotting. Changes in mitochondrial membrane potential were detected by fluorescent imaging and confirmed activation of intrinsic apoptosis in addition to detection of cleaved caspase 9 and 3 but not caspase 8. Reduced cleaved caspase 3 and cleaved PARP was detected in cells with knockdown of the UPR protein CHOP following drug treatment implying an ERS dependent apoptotic process. In addition, mucinous tumor growth in vivo was reduced and ERS stress and apoptosis increased by treatment with orlistat and celecoxib [61].

Additional work from Dilly et al. has utilized a similar in vitro model of mucinous CRC based on a KRAS mutant LS174T MUC2 producing cell line. Mucinous CRC are associated with KRAS mutations, resulting in downstream activation of MAPK and PI3K signaling pathways [12]. MAPK and PI3K signaling has been demonstrated to protect some cancers from ERS cell death [63], leading Dilly et al. to hypothesize that MEK and PI3K inhibition may inhibit survival pathways and sensitize cells to ERS induced apoptosis [64]. Co-treatment of mucinous colon cancer cells with a MEK inhibitor and PI3K inhibitor induced apoptotic cell death as demonstrated by Annexin/PI staining. Dual drug treatment reduced phosphorylated-ERK and phosphorylated-Akt protein levels consistent with MAPK/PI3K signaling inhibition. Expression of PUMA, cleaved caspases 9, cleaved caspase 3 (but not caspase 8), and cleaved PARP increased following dual drug therapy consistent with intrinsic apoptosis signaling. TUNEL assay demonstrated increased apoptosis in tumor explants treated with dual MEK and PI3K inhibition. Dual drug treatment likewise reduced MUC2 secretion in vitro and in explant tissue, which was found to be mediated by significantly reduced FASN expression, an enzyme necessary for MUC2 N-terminal palmitoylation and secretion. The growth of patient-derived xenografts was inhibited by dual MEK and PI3K inhibition [64].

### 6.3. MUC5AC Glycoprotein

In a study by Zhu et al. [65], the expression of MUC5AC was found to be significantly elevated in colon cancer tissues when compared with the corresponding para-cancerous tissues in the clinical setting. In vitro assays showed that inhibition of MUC5AC by siRNA reduced colony formation of the SW620 cell line. Authors transfected a colon cancer cell line with siRNA-control or siRNA-MUC5AC and assessed apoptosis and the cell cycle by flow cytometry. The apoptosis rate was significantly enhanced in MUC5AC knockdown cells. The cell cycle was found to be arrested in G1, but siRNA-control had no effects on the apoptosis and cell cycle of SW620, indicating decreased cell vitality and colony formation were attributed to the MUC5AC-related apoptosis and cell cycle arrest [65]. Upregulation of MUC5AC in CRC cells has recently been confirmed as a mechanism for resistance to 5-FU and oxaliplatin chemotherapy through down-regulation of *p53* and up-regulation of β-catenin [66]. CD44 is a transmembrane glycoprotein involved in several cellular processes like growth and invasion through Src signaling [67]. Pothuraju et al. observed co-localization of MUC5AC and CD44 by immunoprecipitation and confocal microscopy in CRC [66]. Knockdown of MUC5AC decreased cell survival markers including phosphorylated AKT and phosphorylated Src and increased pro-apoptotic cleaved PARP and cleaved-caspase 9. Knockdown of CD44 also decreased expression of Src and AKT. Combination 5FU and oxaliplatin chemotherapy was found to increase MUC5AC expression in a dose-dependent manner in CRC cell lines and cells expressing MUC5AC had greater viability when compared to MUC5AC knockout cells. MUC5AC knockout cells treated with 5FU had higher rates of cell cycle arrest. In addition, *p53* and *p21* expression was increased while Chk1, which mediates p53 activation was reduced. Authors observed that 5FU treatment caused reduced Chk1, leading to cell cycle arrest and subsequent activation of *p53* to induce apoptosis. In addition, 5FU upregulated the Wnt ligand coreceptor Lrp6 which increased expression of β-catenin and its target gene CD44 with authors concluding that MUC5AC permits 5FU resistance through the β-catenin/p53/p21 axis [66].

### 6.4. MUC13 Glycoprotein

Western blot analysis of MUC13 overexpressing cells by Gupta et al. noted that the expression of the anti-apoptotic protein was influenced by MUC13 expression [38]. Sheng et al. explored mechanisms of cell death resistance related to MUC13 expression [68]. In vivo study in mice demonstrated increased intestinal apoptosis in response to DSS exposure in MUC13 knockout mice. In addition, colon cells treated with MUC13 siRNA were more susceptible to apoptosis in response to DSS, UV irradiation and TRAIL [39]. This group subsequently explored expression of regulators of apoptosis and cell death- the BCL2 family and NF-ΚB signalling [68]. In an in vitro CRC model, silencing of MUC13 decreased mRNA expression of the antiapoptotic gene BCL-X_L_. BCL-X_L_ is regulated by NF-kB p65 signalling. Combination of MUC13 knockdown and 5FU chemotherapy caused tumor regression in a CRC xenograft model. Similar to their in vitro findings, siRNA knockdown of MUC13 reduced total NF-κB p65 protein and reduced BCL-X_L_ expression in tumor cells. Interestingly, immunohistochemical staining of colorectal tumors by Evertsson et al. identified association between mucinous histology and NF-κB expression [69].

## 7. Chemotherapy Drug Metabolism and Resistance in Mucinous Colorectal Cancer

Resistance to commonly used chemotherapeutic agents for CRC has been evaluated in mucinous CRC and was found to involve genes responsible for chemotherapy drug metabolism within cancer cells. The expression of recognized drug metabolism genes was compared between 21 patients with mucinous CRC and 30 patients with non-mucinous CRC by Glasgow et al. Mucinous tumors were found to significantly overexpress thymidylate synthase (TYMS) and glutathione S-transferase pi (GSTP1) genes, but no significant difference was noted between the expression of other drug related genes in the study cohort [42]. A recent analysis of The Cancer Genome Atlas (TCGA) dataset identified significant differences in the simple somatic mutation (SSM) rate and copy number variation (CNV) rate in genes with recognized roles in chemotherapy drug resistance in mucinous and non-mucinous CRC [43]. One gene associated with 5-FU resistance, thymidine phosphorylase (TYMP), was found to have an SSM rate of 5.97% in mucinous tumors compared with only 1.08% in non-mucinous tumors. Two genes associated with oxaliplatin resistance were found to be mutated at greater frequencies in the mucinous group when compared with the non-mucinous group. ATPase copper transporting beta (ATP7B) was mutated in 8.96% of mucinous tumors compared with only 3.45% of non-mucinous tumors. Similarly, SRSF protein kinase 1 (SRPK1) was mutated in 10.45% of mucinous tumors compared with only 3.23% of non-mucinous tumors. Two irinotecan resistance-associated genes were mutated at statistically different frequencies between the two cohorts [43]. These findings may be in part responsible for the variation in response to chemotherapy observed in patients with mucinous and non-mucinous CRC.

## 8. Radiotherapy Resistance in Mucinous Colorectal Cancer

A meta-analysis of response to neoadjuvant chemoradiotherapy in mucinous and non-mucinous rectal cancers has identified lower rates of pathological complete response and tumor downstaging in mucinous cases [70]. In addition, a multivariate analysis by Shin et al. indicated that mucinous rectal cancer was a negative prognostic factor for survival following NACRT in rectal cancer [71]. In contrast, analysis of data from the Dutch cancer registry and trial cohorts demonstrated that although mucinous cancers were associated with higher rates of positive circumferential margin positivity, no difference was observed in survival between mucinous rectal and non-mucinous rectal cancers treated with short course radiotherapy (SCRT) or NACRT [72]. It was noted that while survival in mucinous rectal cancer was inferior in the years from 1989 to 1998, that survival was equivalent in the years after 1999 [72]. These findings suggest that margin positivity post treatment may have different implications in mucinous rectal cancer. Mucinous tumors present at the margin may be acellular and due to tumor response [52].

Subsequent to this meta-analysis, a review of the US national cancer database was conducted by Kiran et al. to identify predictors of response to NACRT included 13,742 patients with 32% achieving pCR between 2009–2013. Treatment included NACRT and SCRT. The authors demonstrated that mucinous cases were less likely to achieve pCR [73]. Likewise, Hosseini et al. compared outcomes from 46 mucinous 358 non-mucinous locally advanced rectal cancers undergoing NACRT between 2010–2015. On univariate analysis, mucinous cancer was a poor predictor of pCR [74]. A retrospective review of 151 LARC receiving SCRT by Räsänen et al. indicated increased rates of local recurrence in mucinous rectal cancers [75]. Vernmark et al. specifically studied the impact of SCRT versus NACRT on locally advanced mucinous rectal cancers in a review of 433 patients. NACRT was used more frequently in mucinous rectal cancer compared to non-mucinous rectal cancer. In the mucinous group, no significant differences were found between SCRT and LCRT for survival outcomes [76]. Similarly, a retrospective review by Hammarström found mucinous rectal cancers were more likely to be treated with NACRT or SCRT with additional chemotherapy [77]. There was no difference in pCR rate observed between mucinous and non-mucinous rectal cancers overall [77]. However, no mucinous rectal cancer receiving SCRT alone achieved pCR, but addition of chemotherapy to SCRT improved the pCR rate [77]. Overall, there appears to be a dearth of prospective data comparing radiotherapy approaches for LARC mucinous rectal cancer with evidence heavily reliant on heterogenous database studies.

## 9. Tumor Immune Microenvironment in Mucinous Colorectal Cancer

Chemoresistance of mucinous CRC has been attributed to the abundant extracellular MUC2 protein that may act as a physical barrier to immune infiltration. [29]. Additionally, the mucin layer contains adhesion ligands and suppressive cytokines. This tumor environment may prevent the approach of antigen-presenting cells that are required to initiate an immune response, or effector cells required to kill tumor cells [44]. The immune features of mucinous CRC have been explored to a limited extent in the literature. A study by Tozawa et al. noted peri-tumoral lymphocyte infiltration in just 0–4% of mucinous CRCs compared to 17% of non-mucinous CRCs in a sample of 152 CRCs [78]. However, a recent analysis found no difference in the distribution of stromal CD8+ lymphocytes or tumor CD8+ lymphocytes in CRC with mucinous characteristics [79]. High-density macrophages infiltration has been associated with poor prognosis in solid tumors with M2 differentiated macrophages associated with tumor migration, invasion, and attenuated anti-tumor immunity [80]. A meta-analysis of four studies has demonstrated association of pan-macrophage, M1 macrophage and M2 macrophage infiltration with mucinous CRC [80].

Recent studies have raised an interesting question regarding the immune profile of mucinous CRC and the potential for immune checkpoint inhibition as a therapeutic option. Mismatch-repair deficiency predicts the response of solid tumors to immune checkpoint inhibition and is the basis for the approval of immune checkpoint inhibitors for treatment of MSI high metastatic mCRC [81]. MSI or deficient mismatch repair results in high neoantigen load and increased immunogenicity of MSI tumors. Deficient mismatch repair and MSI high tumors cluster into the consensus molecular subtype (CMS) 1 of CRC, described as the immune subtype due to the high infiltration of lymphocytes [82]. Additionally, despite having a proficient mismatch repair system, some CMS1 tumors contain polymerase (POLE) mutations leading to very high tumor mutational burden and are likely to be susceptible to immunotherapy [83]. Khan et al. compared the distribution of CMS subtypes in mucinous versus non-mucinous CRC in a cohort of 608 patients and determine that 34% of mucinous CRCs were CMS1 subtype compared to 13% of non-mucinous CRC [84]. A higher rate of MSI was also observed in this analysis, consistent with the existing literature [10]. A recent immunohistochemical analysis of CRC tumors showed significant association between mucinous histology and PD-L1 positivity [85]. Llosa et al. investigated the tumor immune microenvironment of mCRC treated with PD-1 blockade in a clinical trial, comparing tumor characteristics of patients who exhibited clinical benefit with patients exhibiting disease progression. Clinical benefit from PD-1 blockade was predicted by a composite score of high extracellular mucin and increased PD-L1 expression at the invasive front [86]. A number of recent studies, albeit in non-CRC models, demonstrated that the MUC1-cystoplasmic subunit activates PD-L1 expression through NK-KB mediated inflammatory signaling and promotes suppression of the tumor immune microenvironment [87,88]. Overall, these findings raise the question of whether ICI may represent a potential treatment option for mucinous CRC given the prevalence of MSI and hypermutation in mucinous CRC.

## 10. Clinical Approach to Mucinous Colorectal Cancer

Surgery a key role in the curative treatment of mucinous CRC. The surgical challenge posed by mucinous tumors is largely restricted to rectal cases due to limited access within the bony pelvis and proximity of adjacent organs. The risk of a positive CRM is higher in mucinous rectal cancer [70], although data do not demonstrate association with increased local recurrence, perhaps due to the presence of acellular mucin rather than tumor cells at the positive margin [70]. Optimizing recognition of a mucinous tumor at preoperative investigations is necessary to plan appropriate neoadjuvant treatment and adequate surgical margins. Surgery according to TME principles or, in advanced cases, beyond the standard TME plane improves chances of obtaining a radical resection in mucinous rectal cancers which are generally larger and less likely to respond to neoadjuvant chemoradiotherapy [72]. Oncological outcomes from open versus laparoscopic resection of mucinous CRC has been explored in a single-center setting by Huang et al. who demonstrated oncological safety of laparoscopy in the era of TME surgery [89]. The preoperative histopathological diagnosis of mucinous rectal cancer is known to be challenging with Sengul et al. reporting 80% of mucinous tumors being missed by biopsy [90]. MRI imaging is diagnostically superior to preoperative biopsy sampling for detection of a mucinous rectal tumor [91]. The characteristic appearance of high signal intensity mucous may be detected using high-resolution T2-weighted MRI and allows accurate differentiation of mucinous and non-mucinous tumors [29,92].

The prognosis of mucinous CRC has been explored in numerous observational studies with mixed results. Overall, the prognostic impact of mucinous CRC appears to be most pronounced in rectal compared to colon cancer and in the metastatic setting. A meta-analysis of 34 studies published in 2012 reported worse OS in mucinous CRC compared to non-mucinous CRC but with significant heterogeneity present in the analysis [93]. Analysis of two large historical databases (SEER 1973–2011, Linköping Cancer Database 1972–2009) published in 2015 demonstrated worse cancer specific survival in mucinous CRC. Multivariate analysis of included cases showed that mucinous status was an independent predictor of poor prognosis in rectal cancer [94]. Most recently, Benesch et al. summarized the clinical, demographic and mortality outcomes from mucinous adenocarcinoma arising in 16 primary sites. Data were derived from the Surveillance, Epidemiology, and End Results Program SEER program, a large US population-level cancer database. The analysis included 448,221 cases of colon cancer (mucinous = 9%) and 194,109 cases of rectal cancer (mucinous = 4.6%). Worse 5-year and 10-year OS was observed in rectal mucinous cancer when compared to non-mucinous rectal cancer (5-year OS = 53.1% vs. 59.8%; 10-year OS = 44.1% vs. 50.7%) [95].

## 11. Future Perspectives: Targeting Cell Death

The literature informs us that mucinous CRC is a distinct form of CRC with molecular characteristics that differentiate it from non-mucinous CRC. It appears that the development of mucinous CRC may diverge from the classic chromosomal instability pathway with elements of microsatellite instability prominent in the molecular characteristics of mucinous CRC. Mucinous CRC is capable of evading cell death, firstly through physical barriers to chemotherapy and secondly by molecular mechanisms to inhibit apoptosis and antagonize chemotherapy drug activity. The current literature identifies a number of relevant questions regarding mucinous CRC and reveals opportunities for future investigation. The interplay between mucinous CRC and the host immune system requires further investigation. Targeted treatment with immune checkpoint inhibitors may be beneficial given the increased association between mucinous tumors and immune checkpoint expression and tumor microenvironment immune suppression. Regular inclusion of extracellular mucin as a variable in chemotherapeutic trials is encouraged to understand the response of mucinous CRC to established and emerging treatments. Although BCL2 inhibitory molecules have had limited effect in preclinical CRC studies due to loss of BCL2 expression [96], emerging BCL-X_L_ inhibitors may represent a targeted approach to mucinous CRC given the increased BCL-X_L_ expression observed in response to MUC1 and MUC5AC overexpression. Pre-clinical data demonstrated the activation of cell survival signaling including Akt and PI3K pathways in response to mucin overexpression. Novel treatment strategies may include inhibition of the PI3K-Akt survival pathway in combination with conventional chemotherapy may impair mucinous cell survival mechanisms. In addition, exploiting MUC2 protein production inherent to mucinous CRC, combination of PI3K-Akt inhibition and induction of ERS stress with molecules such as celecoxib may present a novel strategy to impair MUC2 production, induce ERS stress triggered apoptosis, and prevent cell survival in mucinous CRC. Finally, targeting necroptotic cell death may offer an alternative therapeutic strategy in mucinous tumors with impaired apoptotic pathways.

## Figures and Tables

**Table 1:** Mechanisms for resistance to cell death in mucinous colorectal cancer (CRC).

Mechanism	Effect	Reference
Physical tumor structure	Mucin barrier occluding cells	[29]
	Compressive effect of mucin	
Mucin glycoproteins	Inhibition of mitochondrial apoptotic signaling	[30,31,32]
	Inhibition of death receptor apoptotic signaling.	[33,34,35]
	Downregulation of *p53*	[36,37]
	Activation of JNK1	
	Activation of PI3K/Akt pathway Activation of MAPK pathway	
	Activation of NF-κB pathway	
BCL-2 proteins	Increased expression of BCL-X_L_	[31,37,38]
	Bax G(8) frameshift mutation	[39]
	Increased Livin (IAP) expression	
Death receptors	Reduced Fas ligand expression	[40,41]
	Reduced DR4 expression	
Chemo-resistance genes	Overexpression of TYMS	[42,43]
	Altered mutation rate of genes responsible for chemotherapy metabolism	
Tumor immune microenvironment	Adhesion ligands and suppressive cytokines inhibiting immune cell activity	[44]
